# Loss of RUNX3 expression inhibits bone invasion of oral squamous cell carcinoma

**DOI:** 10.18632/oncotarget.14071

**Published:** 2016-12-21

**Authors:** Junhee Park, Hyun-Jeong Kim, Ki Rim Kim, Sun Kyoung Lee, Hyungkeun Kim, Kwang-Kyun Park, Won-Yoon Chung

**Affiliations:** ^1^ Department of Dentistry, Graduate School, Yonsei University, Seoul 120-749, Republic of Korea; ^2^ Department of Oral Biology, Oral Cancer Research Institute, and BK21 PLUS Project, Yonsei University College of Dentistry, Seoul 120-752, Republic of Korea; ^3^ Department of Dental Hygiene, Kyungpook National University, Sangju 742-711, Korea; ^4^ Department of Applied Life Sciences, Graduate School, Yonsei University, Seoul 120-749, Republic of Korea

**Keywords:** oral squamous cell carcinoma, bone invasion, runt-related transcription factor 3, transforming growth factor-β

## Abstract

High recurrence and lower survival rates in patients with oral squamous cell carcinoma (OSCC) are associated with its bone invasion. We identified the oncogenic role of RUNX3 during bone invasion by OSCC. Tumor growth and the generation of osteolytic lesions were significantly inhibited in mice that were subcutaneously inoculated with RUNX3-knockdown human OSCC cells. RUNX3 knockdown enhanced TGF-β-induced growth arrest and inhibited OSCC cell migration and invasion in the absence or presence of transforming growth factor-β (TGF-β), a major growth factor abundant in the bone microenvironment. RUNX3 knockdown induced cell cycle arrest at the G1 and G2 phases and promoted G2 arrest by TGF-β in Ca9.22 OSCC cells. RUNX3 knockdown also inhibited both the basal and TGF-β-induced epithelial-to-mesenchymal transition by increasing E-cadherin expression and suppressing the nuclear translocation of β-catenin. In addition, the expression and TGF-β-mediated induction of parathyroid hormone-related protein (PTHrP), one of key osteolytic factors, was blocked in RUNX3-knockdown OSCC cells. Furthermore, treating human osteoblastic cells with conditioned medium derived from RUNX3-knockdown OSCC cells reduced the receptor activator of nuclear factor-kappaB ligand (RANKL)/osteoprotegerin ratio compared with treatment with conditioned medium from RUNX3-expressing cells. These findings indicate that RUNX3 expression in OSCC cells contributes to their bone invasion and the resulting osteolysis by inducing their malignant behaviors and production of osteolytic factors. RUNX3 alone or in combination with TGF-β and PTHrP may be a useful predictive biomarker and therapeutic target for bone invasion by oral cancer.

## INTRODUCTION

Oral cancer, a major subtype of head and neck cancers, is one of the 10 most commonly diagnosed cancers, and more than 90% of oral malignancies are squamous cell carcinomas (OSCC) [1, 2]. Despite the improved treatment of OSCC, the 5-year survival rate for oral cancer, approximately 63 percent, is less than the 5-year survival rate for all cancers [3]. Oral cancer readily invades the neighboring jawbone, which increases mortality [4]. When oral cancer directly invades the bone of the maxilla and/or the mandible, patients experience severe dysfunctions in speech, mastication, and/or swallowing and should be treated with a surgical resection of the jawbone, which causes severe physical and psychological problems [5]. In addition, bone invasion is a risk factor for mandibular osteoradionecrosis and thus must be considered when planning radiotherapy [6]. Since bone invasion is closely associated with poor prognosis in oral cancer, finding efficient predictive marker or therapeutic target molecules is required for controlling OSCC bone invasion.

Transforming growth factor-β (TGF-β) is one of the growth factors abundant in the bone microenvironment. This factor is deposited in the bone matrix and released via bone resorption. The TGF-β released due to cancer-induced osteolysis, as well as that released by stromal cells and cancer cells, stimulates tumors to increase the production of osteolytic factors and promote their invasiveness [4, 7, 8]. Although TGF-β has been considered to play dual roles in human cancer progression depending on the cellular context and the tumor stage, i.e., tumorigenic and tumor-suppressive roles [9], a recent study reported that TGF-β signaling may contribute to the bony invasion of oral cancer by modulating receptor activator of nuclear factor-kappaB ligand (RANKL), tumor necrosis factor-α, and connective tissue growth factor expression in oral cancer cells [10].

Runt-related transcription factor 3 (RUNX3) is a functionally important transcription factor of the TGF-β-mediated signaling pathway [11]. RUNX3 acts as a tumor suppressor in many cancers, including stomach, bladder, breast, lung, brain, colorectal, pancreatic, and hepatocellular carcinoma [12, 13]. In these cancers, RUNX3 inhibits oncogenic Wnt signaling by interacting with β-catenin and promotes TGF-β-induced growth inhibition by interacting with SMAD3/SMAD4 [14]. In contrast, RUNX3 reportedly plays an oncogenic role in basal cell carcinoma and epithelial ovarian cancer [13]. In head and neck cancers, the role of RUNX3 is controversial. Several studies have demonstrated that RUNX3 expression correlates with the histologic differentiation grades of OSCCs, and the survival rate of patients with lower RUNX3 expression is significantly decreased compared with that of patients with higher expression [15, 16]. In addition, the inactivation of RUNX3 in OSCCs due to gene promoter hypermethylation is significantly associated with tumor stage and the presence of lymph node metastases [17]. Other studies have indicated an oncogenic function for RUNX3 in head and neck cancer [18]. RUNX3 expression was not observed in normal oral mucosal tissues but was detected in 50% of head and neck cancer tissues. RUNX3 expression correlates well with poor differentiation, invasiveness, metastasis, and resistance to chemotherapeutic drugs [19]. However, the role of RUNX3 in bone invasion via interactions between oral cancer and the bone microenvironment remains unclear.

The present study aimed to determine whether RUNX3 could serve as a predictive marker and/or therapeutic target to treat bone invasion in OSCC. We found that knocking down RUNX3 in Ca9.22 human OSCC cells reduced calvarial osteolysis in mice. This murine model with calvarial injection can be excluded from the stress such as mastication compared with orthotopic mouse models of OSCC and is thereby regarded as a suitable model for OSCC-induced osteolysis [20, 21]. Furthermore, we demonstrate that RUNX3 expression in OSCC cells plays a critical role in bone invasion and the resulting osteolysis by promoting the malignant behaviors of OSCC cells and osteoblastic RANKL expression.

## RESULTS

### RUNX3 knockdown inhibited tumor growth and bone loss in OSCC cell-injected mice

**Figure 1:**
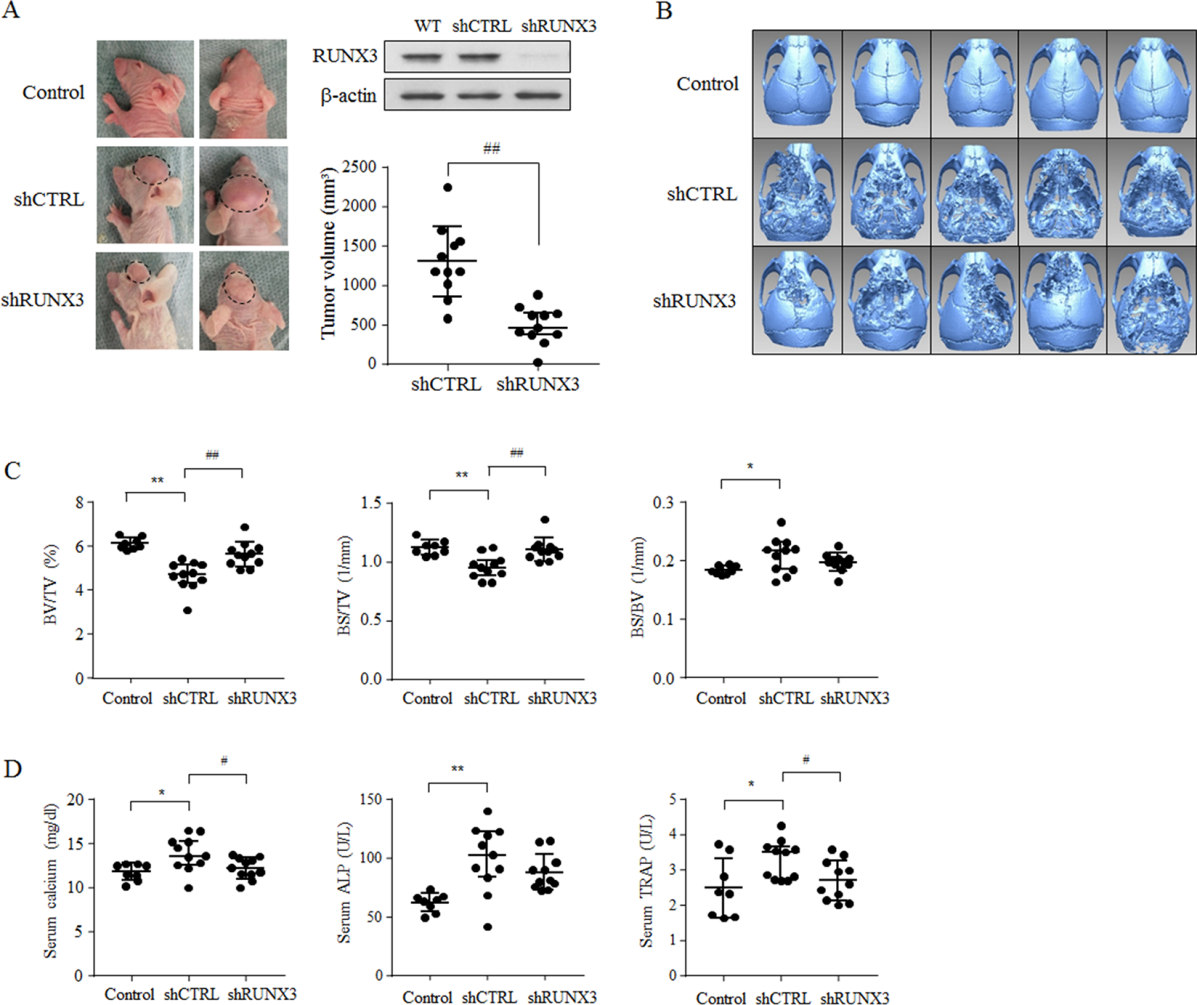

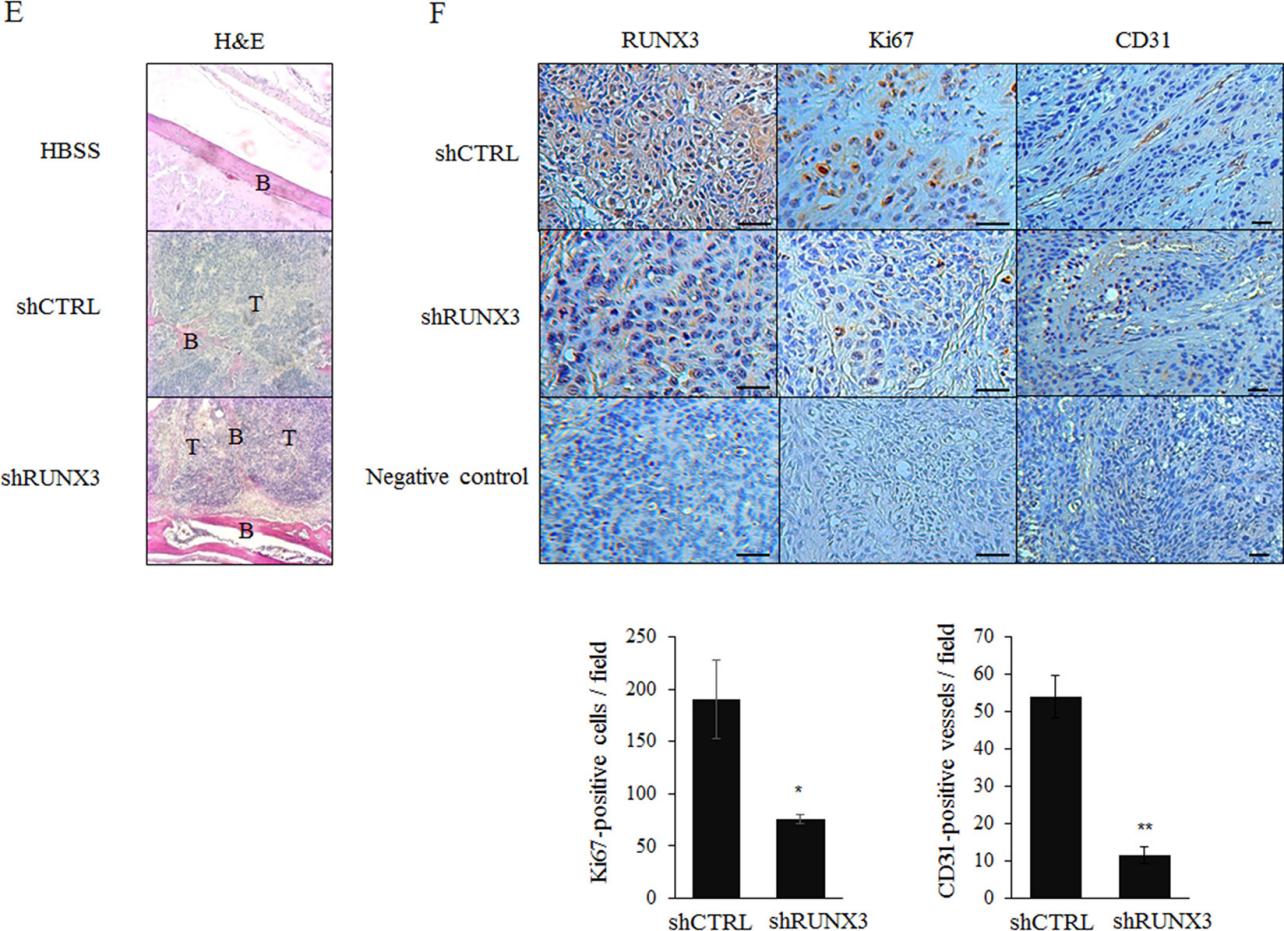
The oncogenic potential of RUNX3 in cancer-induced bone destruction *in vivo* hCTRL or shRUNX3 Ca9.22 OSCC cells (1 × 10^7^ cells/100 μl of HBSS) were subcutaneously injected at the mouse calvaria (*N* = 11). Control mice (*N* = 9) were injected with HBSS only. (**A**) RUNX3 expression level in wild type (WT), shCTRL, and shRUNX3 Ca9.22 cells was detected with a Western blot analysis with its specific primary antibody. On day 28, the tumor volumes were measured. (**B**) On day 28, two-dimensional (2D) images of the collected carvaria were generated from the μCT data using the NRrecon software, and 3D images were reconstructed from 2D images with the rapidform2006 software. (**C**) BV/TV (%), BS/TV (1/mm), and BS/BV (1/mm) served as bone morphometric parameters of the calvaria were determined using the μCT images. (**D**) Serum levels of the bone turnover markers Ca^2+^, ALP, and TRAP5b were estimated using kits as described in the Materials and Methods. (**E**, **F**) The calvarial tissues were fixed with 1% buffered formalin, decalcified in 10% EDTA solution and sectioned. The sections were stained with H&E (original magnification, 100×) (E) and immunostained with specific antibodies against RUNX3, CD31, and Ki67 (original magnification, 200×) (F). Scale bar = 100 μm. Proliferative index and microvessel density were evaluated by immunostaining for Ki67 and CD31, respectively. The images are representative of two independent experiments. The results are combined data from two independent experiments and expressed as the median with interquartile range of 9 or 11 mice per group. *P < 0.05, ^*^P < 0.005 versus HBSS-injected control mice, ^#^*P* < 0.05, ^##^*P* < 0.005 versus shCTRL cell-inoculated mice.

To examine the correlation of RUNX3 expression in oral cancer cells with tumor growth and cancer-induced bone destruction, we first established stable RUNX3-knockdown (shRUNX3) Ca9.22 cells and its control (shCTRL) cells by transducing lentiviral particles with RUNX3 and non-specific short hairpin RNAs, respectively. The combined *in vivo* data were derived from two independent experiments (Supplementary Figure S1). Tumor growth was significantly inhibited by 63% in mice that were subcutaneously injected with shRUNX3 cells at the calvaria compared with mice inoculated with shCTRL cells (Figure 1A). The three-dimensional (3D) images from the μCT data showed that inoculation with shCTRL cells induced severe bone destruction, but RUNX3 knockdown inhibited bone destruction (Figure 1B). Among the values of the bone morphometric parameters, the bone volume/tissue volume (BV/TV, %) and bone surface/tissue volume (BS/TV, 1/mm) were significantly decreased and the bone surface/bone volume (BS/BV, 1/mm) was increased in shCTRL cell-injected mice compared with control mice. BV/TV is one of the most important in revealing the microstructure of cancellous bone. BS/TV and BS/BV indicate bone surface density and bone-specific surface, respectively. RUNX3 knockdown recovered these values to almost control levels although the BS/BV value did not show the statistical significance between shCTRL and shRUNX3 cell-injected mice (Figure 1C). The serum levels of the bone metabolism markers calcium, tartrate-resistant acid phosphatase (TRAP), and alkaline phosphatase (ALP) were also higher in shCTRL cell-inoculated mice than in control mice. The levels of serum calcium and TRAP were inhibited significantly by RUNX3 knockdown. The serum ALP level was also decreased in shRUNX3 cell-injected mice but not significantly different between shCTRL and shRUNX3 cell-injected mice (Figure 1D). Hematoxylin and eosin (H&E) staining indicated that bone was intermingled with tumor due to aggressive tumor growth and serious bone loss in shCTRL cell-injected mice, whereas a broad tumor front and clear interface between the bone and tumor were observed in shRUNX3 cell-injected mice (Figure 1E). The immunohistochemical analysis revealed that Ki67 as a proliferation marker and CD31 as an endothelial cell marker were highly expressed in the tumor tissues of shCTRL cell-injected mice, but RUNX3 knockdown decreased the expression levels of these markers (Figure 1F). These results demonstrate that RUNX3 may be an oncogenic protein in Ca9.22 OSCC cells and play a part in oral cancer-induced bone destruction *in vivo*.

### RUNX3 knockdown inhibited the malignant behaviors of oral cancer cells

**Figure 2:**
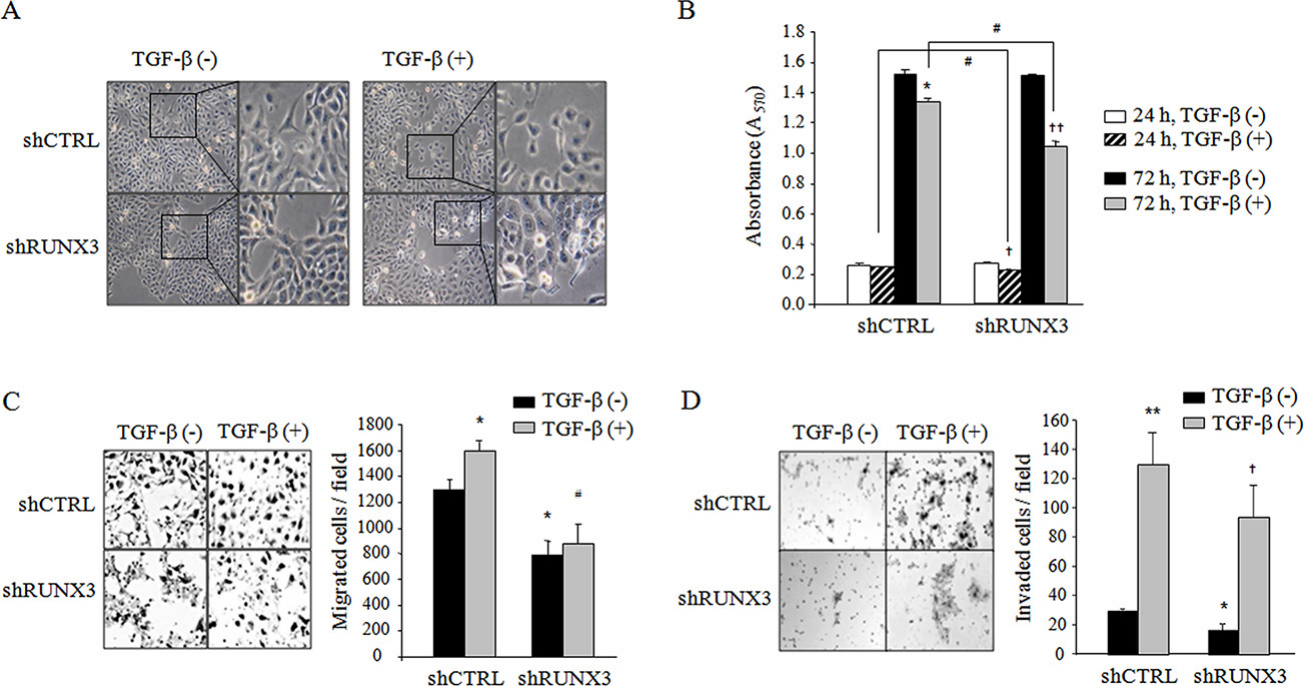
The effect of RUNX3 knockdown on the viability, migration, and invasion of OSCC cells in the absence or presence of TGF-β (**A**) shCTRL or shRUNX3 Ca9.22 cells (1 × 10^3^ cells/well) were treated with 10 ng/ml TGF-β. Cells were observed with an inverted microscope after 24 h of incubation (original magnification, 100×), and (**B**) viability was determined using an MTT assay for cells treated for 24 h or 72 h. (**C**) Lower surface of the membrane was coated with gelatin (1 mg/ml in distilled water), and shCTRL and shRUNX3 Ca9.22 cells (1 × 10^4^ cells/0.1 ml) were seeded into the upper chamber. (**D**) After the lower and upper surfaces of membrane were coated with gelatin and Matrigel (1 mg/ml in distilled water), respectively, shCTRL and shRUNX3 Ca9.22 cells (5 × 10^4^ cells/0.1 ml) were seeded into the upper chamber. Complete medium containing 5% FBS and 10 ng/ml TGF-β was added to the upper chamber, and the lower chamber was filled with 0.6 ml of complete medium containing 10% FBS. After a 24-h incubation, the migrated or invaded cells were fixed and stained with hematoxylin. The number of migrated or invaded cells was counted and imaged using a Zeiss Axio imager microscope (original magnification, 200×). The results are expressed as the mean ± SE. *P < 0.05, ^*^P < 0.01 versus shCTRL cells, ^#^*P* < 0.05 versus TGF-β-treated shCTRL cells, ^†^*P* < 0.05, ^††^*P* < 0.001 versus shRUNX3 cells.

Next, we investigated the possible link between RUNX3 expression and the malignant behaviors of OSCC cells. Noticeable morphological changes were not detected, but increased cell-cell contacts were observed in shRUNX3 cells compared with shCTRL Ca922 cells. TGF-β treatment reduced cell-cell contact in shCTRL cells but not in shRUNX3 cells (Figure 2A). Culturing shCTRL and shRUNX3 cells in the absence of TGF-β for 24 h and 72 h did not result in significant differences in the viabilities of these cells. RUNX3 knockdown inhibited cell viability by 12% and 23% in Ca9.22 cells stimulated with TGF-β for 24 h and 72 h (Figure 2B). In the absence of TGF-β, RUNX-3 knockdown reduced cell migration and invasion by 38% (Figure 2C) and by 44% (Figure 2D), respectively. TGF-β stimulation for 24 h significantly increased the migration of shCTRL cells, not shRUNX3 cells (Figure 2C) and promoted the invasion of shCTRL and shRUNX3 cells (Figure 2D). RUNX3 knockdown significantly inhibited TGF-β-induced cell invasion (Figure 2D). Furthermore, we investigated the effect of RUNX3 knockdown in YD10B cells, another RUNX3-expressing OSCC cells (Supplementary Figure S2A). RUNX3 knockdown did not affect the viability (Supplementary Figure S2B). TGF β stimulation increased cell invasion by 23% in RUNX3-expressing siCTRL cells and by 11% in RUNX3 knockdown siRUNX3 cells. RUNX3 knockdown remarkably inhibited cell invasion in the absence or presence of TGF-β in YD10B cells (Supplementary Figure S2C). These results indicate that RUNX3 expression is associated with the malignant behavior of OSCC cells.

### RUNX3 knockdown promoted TGF-β-induced cell cycle arrest in Ca9.22 oral cancer cells

To further examine the effect of RUNX3 expression and TGF-β stimulation on the growth of Ca9.22 cells, the cell cycle distributions of shCTRL and shRUNX3 cells were analyzed after treatment with TGF-β for 24 h. Compared with shCTRL cells, the cell populations in the sub-G1 and S phase were reduced, and those in G1 and G2 phase were significantly increased in shRUNX3 cells, irrespective of TGF-β treatment. TGF-β stimulation significantly increased the population of shRUNX3 cells in the G2 phase (Table 1). These results suggest that RUNX3 knockdown induced cell cycle arrest in the G1 and G2 phases in Ca9.22 OSCC cells.

**Table 1:** The effect of RUNX3 knockdown on the cell cycle distribution of oral cancer cells

Cell cycle	shCTRL cells		shRUNX3 cells	
	TGF-β (–)	TGF-β (+)	TGF-β (–)	TGF-β (+)
Sub-G1	3.1 ± 0.2	3.7 ± 0.5	2.2 ± 0.1*	2.6 ± 0.2
G1	42.9 ± 0.9	42.3 ± 0.8	47.2 ± 0.5*	46.2 ± 0.3^#^
S	43.4 ± 0.6	43.6 ± 0.3	39.1 ± 0.4*	38.1 ± 0.1^#^
G2	10.9 ± 0.04	11.7 ± 0.6	11.6 ± 0.2*	13.8 ± 0.2^#,†^

shCTRL and shRUNX3 Ca9.22 cells (1 × 10^5^ cells/dish) were treated with 10 ng/ml TGF-β for 24 h. Cell cycle analysis was performed using a FACSverse cytometer. Results are expressed as the mean ± S.E. *P < 0.05 versus shCTRL cells, ^#^*P* < 0.05 versus TGF-β-treated shCTRL cells, ^†^*P* < 0.0.5 versus shRUNX3 cells.

### RUNX3 knockdown inhibited the epithelial-to-mesenchymal transition (EMT) of OSCC cells

**Figure 3:**
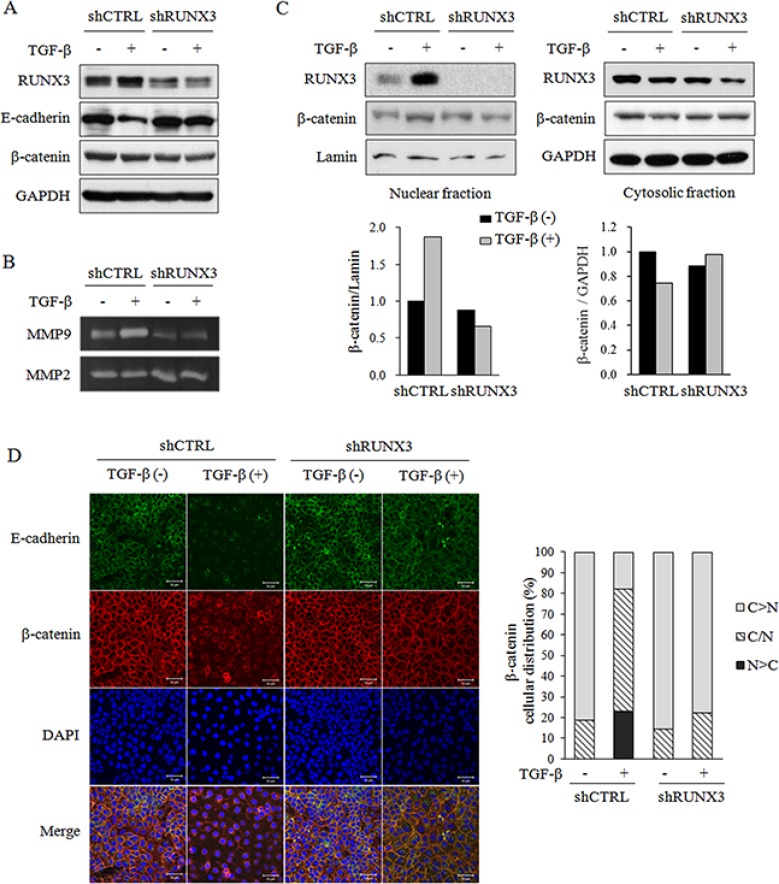
The effect of RUNX3 knockdown on the expression of EMT markers by OSCC cells in the absence or presence of TGF-β shCTRL or shRUNX3 Ca9.22 cells (1 × 10^5^ cells/dish) were treated with 10 ng/ml TGF-β for 24 h. (**A**) The expression levels of RUNX3, E-cadherin, and β-catenin were examined in the total lysates with a Western blot analysis. GAPDH served as a loading control. (**B**) Conditioned media were collected, and the MMP-2 and MMP-9 activities were determined using gelatin zymography. (**C**) Cytoplasmic and nuclear protein extracts were prepared using a nuclear/cytosol fractionation kit. The levels of RUNX3 and β-catenin in each fraction were determined via Western blot analysis. Lamin served as a loading control for the nuclear fraction. The images are representative of three independent experiments. The graphs illustrate the ratio of the densitometric intensity of β-catenin after normalization to lamin or GAPDH. (**D**) Subcellular localization of E-cadherin and β-catenin was examined by confocal microscopy. The cells were double immunostained for E-cadherin (green) and β-catenin (red) and co-stained with DAPI for DNA. The fields shown were independently visualized by confocal microscopy, and three images were then overlaid (Merge). Cellular distribution of β-catenin was shown as percentage of shCTRL or shRUNX3 cells with its cytoplasmic (C > N), cytoplasmic and nuclear (C/N), and nuclear (N > C) localization.

EMT is a process whereby epithelial cells are transcriptionally reprogrammed, resulting in decreased adhesion and enhanced migration or invasion [22, 23]. TGF-β also increases OSCC cell invasion by regulating EMT markers and stimulating the production of factors that prolong osteoclast survival [24]. To determine whether the decreases in migration and invasion were due to the inhibition of EMT in TGF-β-treated and untreated shRUNX3 Ca9.22 cells, we examined the expression levels of EMT markers. Western blot analysis indicated that TGF-β stimulation increased RUNX3 expression. The expression of E-cadherin, an epithelial marker, was increased in shRUNX3 cells and its reduced expression by TGF-β in shCTRL was not detected in shRUNX3 cells. RUNX3 knockdown did not affect the cellular level of β-catenin which is a mesenchymal marker (Figure 3A). Matrix metalloproteinase (MMP)-9 levels in the culture media were elevated in TGF-β-treated shCTRL cells but was decreased in shRUNX3 cells, whereas the MMP-2 levels in the culture media were not influenced by RUNX3 knockdown or TGF-β treatment (Figure 3B). The translocation of β-catenin to the nucleus was promoted by TGF-β stimulation in shCTRL cells but was not increased in TGF-β-stimulated shRUNX3 cells (Figure 3C). Immunofluorescence images also showed that E-cadherin expression was decreased and the number of cells with the nuclear β-catenin was increased by TGF-β stimulation in shCTRL cells. The effect of TGF-β on E-cadherin and the nuclear translocation of β-catenin was not detected in shRUNX3 cells. E-cadherin and β-catenin were mainly detected along the membranes in shRUNX3 cells (Figure 3D). We further confirmed the effects of RUNX3 knockdown and TGF-β stimulation on EMT-related markers in YD10B cells. E-cadherin expression was increased and not decreased by TGF-β stimulation in RUNX3 knockdown YD10B cells (Supplementary Figure S3). RUNX3 knockdown inhibited the cellular level (Supplementary Figure S3A) and the nuclear translocation (Supplementary Figure S3B) of β-catenin, irrespective of TGF-β-stimulation. Immunofluorescence images supported that the effect of TGF-β on E-cadherin and β-catenin was also blocked by RUNX3 knockdown in YD10B OSCC cells (Supplementary Figure S3C). These results suggest that RUNX3 expression contributes to the induction of EMT in OSCC cells.

### RUNX3 knockdown inhibited parathyroid hormone-related protein (PTHrP) expression in OSCC cells and RANKL expression in osteoblasts

**Figure 4:**
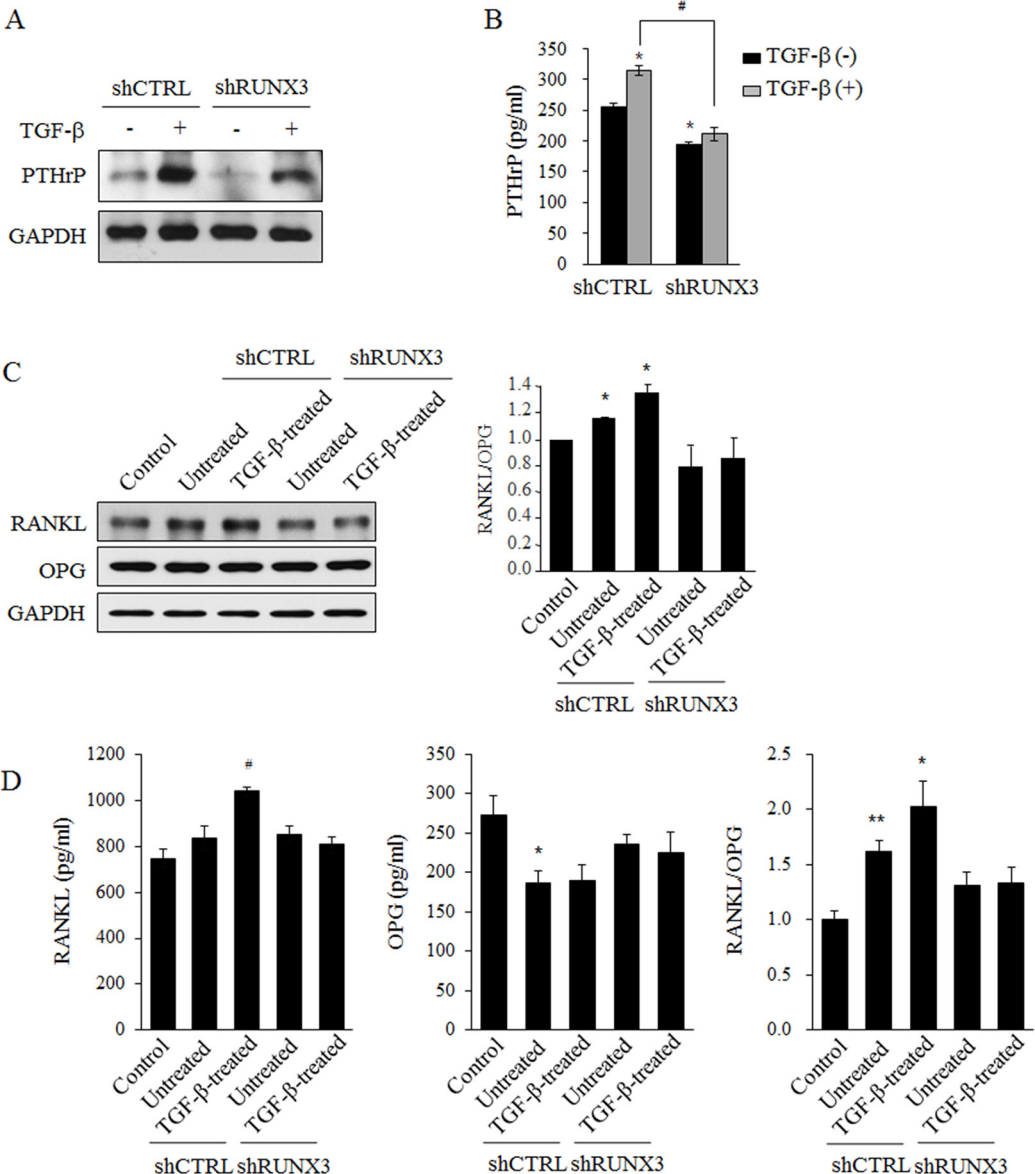
The effect of RUNX3 knockdown on the expression of PTHrP in OSCC cells and RANKL expression in osteoblasts (**A**) shCTRL or shRUNX3 Ca9.22 cells (1 × 10^5^ cells/dish) were treated with 10 ng/ml TGF-β for 24 h. The expression level of PTHrP was examined in the total lysates with a Western blot analysis. GAPDH served as a loading control. (**B**) shCTRL or shRUNX3 Ca9.22 cells (1 × 10^3^ cells/well) were treated with 10 ng/ml TGF-β for 24 h. PTHrP levels in the conditioned media were analyzed with a ELISA kit. The results are expressed as the mean ± SE. *P < 0.05 versus shCTRL cells without TGF-β. (**C**) hFOB1.19 osteoblastic cells (1 × 10^6^ cells/dish) were incubated with the respective shCTRL or shRUNX3 Ca9.22 cell-derived conditioned media for 6 h. The expression levels of RANKL and OPG in osteoblastic cells were determined with a Western blot analysis. GAPDH served as a loading control. (**D**) hFOB1.19 cells (1 × 10^4^ cells/well) were incubated for 24 h with or without conditioned media derived from shCTRL or shRUNX3 Ca9.22 cells. The culture media from hFOB1.19 cells were analyzed for the secreted levels of RANKL and OPG with ELISA kits. The results are expressed as the mean ± SE. *P < 0.05 versus control (without conditioned media), ^#^*P* < 0.05 versus conditioned media derived from untreated shCTRL cells.

Cancer-induced bone destruction is due to the action of osteoblasts and osteoclasts rather than the direct action of tumor cells. RANKL and its decoy receptor osteoprotegerin (OPG) are key factors in cancer-induced bone destruction. These factors modulate the differentiation and activation of osteoclasts, and their expression is regulated by cancer cell-derived osteolytic cytokines [4]. PTHrP has been recognized as a major osteolytic cytokine whose expression is induced by TGF-β during cancer-induced bone destruction [25]. Western blot analysis indicated that TGF-β stimulation upregulated PTHrP expression in Ca9.22 (Figure 4A) and YD10B cells (Supplementary Figure S4A) but RUNX3 knockdown inhibited PTHrP expression in these two OSCC cells stimulated with TGF-β or not. In addition, TGF-β stimulation significantly increased PTHrP levels in the culture media of RUNX-3 expressing OSCC cells and TGF-β-induced PTHrP secretion was blocked RUNX3 knockdown (Figure 4B, Supplementary Figure S4B) Furthermore, RANKL expression (Figure 4C) and secretion (Figure 4D) in human hFOB1.19 osteoblastic cells increased by treatment with shCTRL cell-derived conditioned medium and increased further when the cells were treated with conditioned medium of TGF-β-stimulated shCTRL cells. In contrast, conditioned media of shRUNX3 cells or TGF-β-stimulated shRUNX3 cells did not affect RANKL expression and secretion in osteoblasts. Conditioned media of both TGF-β-treated and untreated shCTRL and shRUNX3 cells did not affect OPG expression. However, the secreted OPG levels were reduced by conditioned media of both TGF-β-treated and untreated shCTRL but these reduction was blocked by RUNX3 knockdown (Figure 4D). These findings suggest that RUNX3 expression promotes PTHrP production in OSCC cells and thereby induces RANKL production and increased RANKL/OPG ratio in osteoblasts.

### RUNX3 gene expression enhanced in head and neck squamous cell carcinoma (HNSCC)

**Figure 5:**
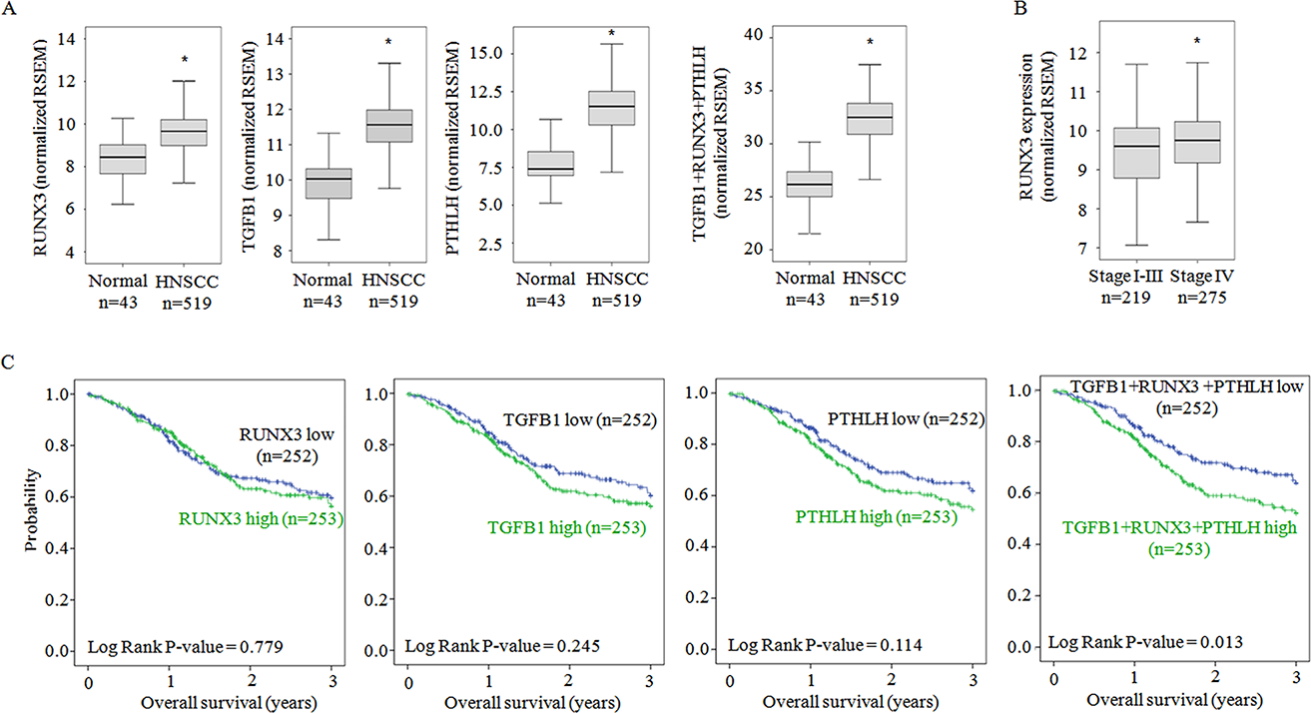
The relationship between oral cancer and the expression levels of RUNX3, TGF-β and PTHrP (**A**) Box and whisker plots show the expression level of RUNX3, TGF-β and PTHrP. Box represents the median and the quartiles. Whisker expresses 1.5 interquartile range (IQR) of the lower or the upper quartile. (**B**) The expression level of RUNX3 grouped by tumor stage was examined. (**C**) Kaplan–Meier survival curves show the probability of overall survival in relation to the expression of RUNX3, TGF-β, PTHrP and the combination of expression level of three genes in patients with head and neck cancer. For each gene, the head and neck cancers were classified into high- or low-expressing groups according to whether the expression of the candidate gene was greater than the median expression of the gene. *P < 0.001 versus normal.

To estimate the clinical significance of RUNX3 expression, we analyzed The Cancer Genome Atlas (TCGA) microarray data. RUNX3 gene expression was significantly higher in HNSCC tissues than in normal tissues (Figure 5A) and in the most advanced stage IV with bone invasion than in other stages (Figure 5B). The gene expressions of TGF-β as a RUNX-regulating growth factor and PTHrP as a RUNX3-regulated osteolytic cytokine were also upregulated in HNSCC tissues than normal tissues (Figure 5A). In Kaplan-Meier survival analysis, high gene expression of RUNX3 in combination with those of TGF-β and PTHrP was significantly correlated with poor overall survival (Figure 5C).

## DISCUSSION

Bone invasion is a common characteristic of OSCC. In particular, gingival squamous cell carcinomas invade the mandible in 12 to 56% of patients to worsen the patient's prognosis and finally cause death [26, 27]. Osteoclasts, rather than the carcinomas themselves, have been recognized as key players in bone destruction due to cancer invasion [28]. Thus, a number of clinical trials have focused on targeting factors derived from bone metabolism rather than tumor-derived factors [29]. However, novel cancer-specific biomarkers are required to detect bone invasion early and control the growth and invasive capacity of cancer cells, which modulate bone microenvironment by releasing osteolytic factors and ultimately cause osteoclast activation. Such biomarkers may improve clinical outcomes. Therefore, we investigated the potential of RUNX3 as a cancer-specific marker for OSCC-mediated bone invasion.

We identified the oncogenic role of RUNX3 in the growth and bone invasion of OSCC. The bone invasion and resultant osteolysis as well as the tumor volume were noticeably reduced in mice inoculated with Ca9.22 OSCC cells in which RUNX3 was knocked down, as supported by histological examination, bone morphometric μCT analyses, and the detection of bone turnover markers in the sera. Histologically, oral cancer is associated with two types of bone invasion [30]. The erosive type is characterized by a broad tumor front and sharp interface between the bone and tumor, whereas the infiltrative type is characterized by an irregular interface and finger-like projections of oral cancer into the bone. The median disease-free survival is 5.5 years for OSCC patients with erosive bone invasion but 1.5 years for patients with bone invasion [31]. Interestingly, Ca9.22 OSCC cells infiltrated the bone, but this bone invasion was inhibited by RUNX3 knockdown.

We attempted to verify the mechanism underlying the oncogenic role of RUNX3 in the bone invasion of OSCC cells. During the initial phase of bone invasion, cancer cells produce proteases to degrade the extracellular matrix and facilitate the entry of cancer cells into the soft tissues or marrow spaces within the bone. The cancer cells then release cytokines to directly or indirectly induce excessive osteoclastogenesis. Finally, osteoclast-mediated bone resorption results in the release of several growth factors, and these factors stimulate cancer cells [20]. Among these growth factors, TGF-β has been considered essential to the bony invasion of oral cancer [10]. In our study, RUNX3 knockdown and TGF-β stimulation resulted in the distinctive responsiveness to TGF-β in the viability of Ca9.22 and YD10B OSCC. However, RUNX3 knockdown reduced noticeably the invasive capacity of both OSCC cells in the absence or presence of TGF-β, as supported by the enhanced E-cadherin expression and the reduced nuclear level of β-catenin compared with RUNX3-expressing cells. These changes in the expression levels of EMT markers are consistent with the reduced invasion in RUNX3-knockdown OSCC cells.

To investigate the mechanism by which RUNX3 expression in OSCC cells could cause osteolysis, we focused on the role of RUNX3 and TGF-β in the interactions between oral cancer and the bone microenvironment. RUNX3 knockdown inhibited PTHrP production and the induction of PTHrP by TGF-β in OSCC cells. Moreover, RUNX3 knockdown in OSCC cells inhibited osteoblastic RANKL production. These results suggest that the production of PTHrP via RUNX3 in OSCC mediates osteoblastic RANKL and OPG production. The enhanced RANKL expression and RANKL/OPG ratio are well-known factors that promote bone resorption by triggering osteoclast formation [32–34].

Finally, the gene expression levels of RUNX3, TGF-β (a RUNX3-regulating growth factor), and PTHrP (a RUNX3-regulated osteolytic factor) were higher in the tissues of patients with head and neck cancer, including oral cancers, than in normal tissues. The increased RUNX3 gene expression in the most advanced tumor stage, stage IV, with bone invasion is consistent with our *in vitro* and *in vivo* data suggesting the oncogenic role of RUNX3 in oral cancer bone invasion.

**Figure 6:**
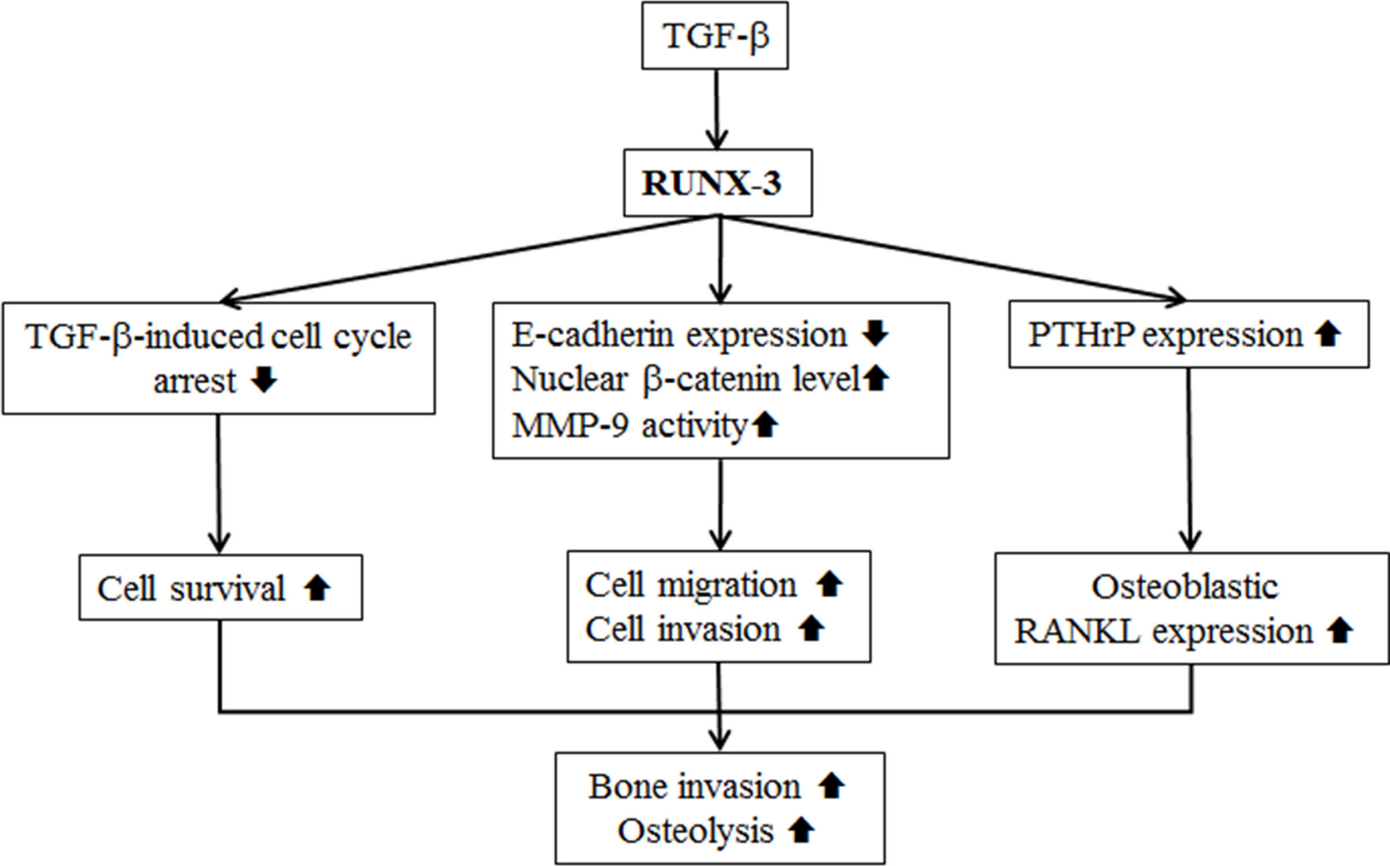
The oncogenic role of RUNX3 in the bone invasion of Ca9.22 OSCC cells is summarized

In summary, RUNX3 participates in cancer-induced bone destruction by affecting the survival, migration, invasion, and/or TGF-β responsiveness of OSCC cells and by increasing the production of PTHrP by OSCC cells (Figure 6). RUNX3 independently or in combination with TGF-β and PTHrP may serve as a biomarker for predicting the prognosis of and potential for bone invasion in oral cancer patients, and it may also serve as a potential therapeutic target for controlling cancer invasion.

## MATERIALS AND METHODS

### Reagents and antibodies

Dulbecco's modified Eagle's medium (DMEM), Ham's F-12 nutrient mixture, Dulbecco's modified Eagle's medium nutrient mixture F-12 (DMEM/F-12) without phenol red, fetal bovine serum (FBS), Hank's balanced salt solution (HBSS), phosphate-buffered saline (PBS), antibiotic-antimycotic mixture containing 100 U/ml penicillin and 100 μg/ml streptomycin, Geneticin^®^ (G418), and 0.25% trypsin-EDTA were purchased from Gibco BRL (Grand Island, NY, USA). Recombinant human TGF- β1 was obtained from Millipore (Billerica, MA, USA). Cholera toxin, hydrocortisone, insulin, apo-transferrin, triiodothyronine (T3), 3-(4,5-dimethylthiazol-2yl)-2,5-diphenyl tetrazolium bromide (MTT), and propidium iodide (PI) were purchased from Sigma-Aldrich Chemical (St. Louis, MO, USA). The antibodies were obtained from the following sources: polyclonal anti-rabbit antibodies against E-cadherin, β-catenin, RANKL, and PTHrP, monoclonal anti-mouse antibodies against OPG and glyceraldehyde 3-phosphate dehydrogenase (GAPDH), and horseradish peroxidase (HRP)-conjugated secondary antibodies (Santa Cruz Biotechnology, Santa Cruz, CA, USA); anti-mouse E-cadherin monoclonal antibody (BD Biosciences, San Jose, CA, USA); anti-mouse RUNX3 monoclonal (catalog #ab40278), anti-rabbit CD31 polyclonal (ab28364), and anti-rabbit Ki67 polyclonal (catalog #ab15580) antibodies (Abcam, Cambridge, UK); anti-rabbit β-actin polyclonal antibody (Sigma-Aldrich); and HRP-goat anti-mouse/anti-rabbit IgG (H+L, Invitrogen, Carlsbad, CA, USA). All reagents used in this study were of analytical grade.

### Cell culture

Ca9.22 cells derived from human gingival squamous cell carcinoma were purchased from the Japanese Collection of Research Bioresources Cell Bank (Shinjuku, Japan), and the cells were grown in DMEM/F12 (3:1 ratio) medium supplemented with 10% FBS, 1 × 10^–10^ M cholera toxin, 0.4 mg/ml hydrocortisone, 5 μg/ml insulin, 5 μg/ml apo-transferrin, and 2 × 10^–11^ M T3 in a humidified atmosphere of 5% CO_2_ at 37°C. hFOB1.19 human fetal osteoblastic cells were cultured in DMEM/F12 without phenol red with 10% FBS, 1% antibiotic-antimycotic mixture and 0.3 mg/ml G418 at 34°C under humidified atmosphere of 5% CO_2_.

### Animals

Five-week-old male Balb/c *nu/nu* mice (19 ± 1 g) were obtained from the NARA Biotech (Seoul, Korea) and acclimatized for 1 week. Mice were provided access to a commercial rodent chow and tap water *ad libitum* and housed under specific pathogen-free conditions with a relative humidity of 50 ± 5% and a 12-h light/dark cycle at 22 ± 2°C. All animal experiments were approved by the Institutional Animal Care and Use Committee of the Department of Laboratory Animal Resources, Yonsei Biomedical Research Institute, Yonsei University College of Medicine (Approval number: 2013–0066).

### RUNX3 knockdown

Ca9.22 cells were transduced with RUNX3 short hairpin RNA (shRNA) lentiviral particles (Santa Cruz Biotechnology, Santa Cruz, CA, USA) (shRUNX3 cells) or non-specific shRNA lentiviral particles (Santa Cruz Biotechnology) (shCTRL cells) as a control. Briefly, Ca9.22 cells were seeded at 1 × 10^5^ cells per 60-mm dish 24 h prior to viral infection. The cells were then infected with viral supernatants in the presence of 10 μg/ml polybrene for 24 h. The infected cells were maintained in fresh complete medium for 24 h and then cultured in complete medium containing puromycin (10 μg/ml) for an additional 2 weeks. Puromycin-resistant clones were selected and expanded. The level of RUNX3 in shCTRL and shRUNX3 Ca9.22 cells was determined via Western blot analysis.

### A murine calvarial model for cancer-induced bone destruction

shCTRL or shRUNX3 Ca9.22 cells (1 × 10^7^ cells/ 100 μl of HBSS) were subcutaneously injected over the calvaria of mice (*n* = 5 or 6) using a 1-ml syringe with a sterile 26-gauge needle. The control group (*n* = 3 or 6) was injected with only HBSS. On day 28, the tumor volumes were measured using an electric digital caliper and calculated according to the following formula: tumor volume (mm^3^) = (a × b^2^)/2, where ‘a’ is the longest diameter and ‘b’ is the shortest diameter of the tumor. The mice were anesthetized by intraperitoneal injection of 30 mg/kg Zoletil (Virbac Laboratories, Carros, France) and 10 mg/kg Rompun (Bayer HealthCare Korea, Seoul, Korea) mixture. The blood was collected by cardiac puncture and the calvaria were collected after cervical dislocation.

The collected calvaria were analyzed scanned with a μCT system (SkyScan 1076, SkyScan, Aartselaar, Belgium) as described previously [35]. To quantitatively analyze bone destruction, the bone volume/tissue volume (BV/TV, %), bone surface/tissue volume (BS/TV, 1/mm), bone surface/bone volume (BS/BV, 1/mm) within the volume of interest (VOI) were obtained using the CTAn software (SkyScan).

The collected blood samples were clotted at room temperature for 2 h and centrifuged at 900 × g for 20 min to obtain sera. The serum levels of calcium and alkaline phosphatase (ALP) were measured using a QuantiChrome Calcium and ALP assay kit (BioAssay Systems, Hayward, CA, USA) and the serum level of tartrate-resistant acid phosphatase (TRAP) 5b was determined with a mouse TRAP assay kit (Immunodiagnostic Systems, Boldon, UK) according to the manufacturer's instructions.

Paraffin-embedded calvarial tissues were serially sectioned (4-μm thick), mounted on slides, deparaffinized in xylene, rehydrated, and stained with hematoxylin and eosin (H&E). The stained tissues were then dehydrated and mounted with Shandon Synthetic Mountant solution (Thermo Scientific, Waltham, MA, USA). For the immunohistochemical examination, the rehydrated sections were treated with Digest-All 3 pepsin solution (Life Technologies, Grand Island, NY, USA) for 20 min at 37°C, 3% hydrogen peroxide for 15 min at room temperature, and goat serum for 30 min. The sections were incubated with a 1:100 dilution of each primary antibody against RUNX3, CD31, and Ki67 at 4°C overnight and then treated with HRP-conjugated secondary antibodies diluted 1:200 for 1 h at room temperature; the HRP activity was then determined using a DAB system (Lab Vision, Fremont, CA, USA). The sections were counterstained with Mayer's hematoxylin, dehydrated, mounted, and observed under a Zeiss Axio imaging microscope.

### Cell viability assay

shCTRL and shRUNX3 Ca9.22 cells (1 × 10^3^ cells/well) were seeded into a 96-well plate and incubated in complete media for 24 and 72 h with or without TGF-β (10 ng/ml). Cell viability was measured with an MTT assay (Jun et al., 2014).

### Transwell migration and invasion assay

The migratory and invasive ability of shCTRL and shRUNX3 Ca9.22 cells was determined using a 6.5-mm transwell chamber with an 8.0-μm pore polycarbonate membrane (Corning Costar, Lowell, MA). For the migration assay, the lower surface of membrane was coated with gelatin (1 mg/ml in distilled water), and shCTRL and shRUNX3 Ca9.22 cells (1 × 10^4^ cells/0.1 ml) were seeded into the upper chamber. For the invasion assay, the lower and upper surfaces of the membrane were coated with gelatin and Matrigel (1 mg/ml in distilled water), respectively. shCTRL and shRUNX3 Ca9.22 cells (5 × 10^4^ cells/0.1 ml) were seeded into the upper chamber. The complete medium containing 5% FBS and 10 ng/ml TGF-β was added to the upper chamber, and the lower chamber was filled with 0.6 ml of complete medium containing 10% FBS. After a 24-h incubation, the number of the migrated or invaded cells was counted as described previously [35].

### Cell cycle distribution analysis

shCTRL and shRUNX3 cells (1 × 10^5^ cells/dish) were seeded in 100-mm culture dishes, and attached cells were treated with 10 ng/ml TGF-β for 24 h. The cells were trypsinized, resuspended in PBS, and fixed in 70% ethanol for 12 h. The cells were then stained with 0.1% (v/v) Triton X-100, 0.02 mg/ml PI, and 0.1 mg/ml RNase A for 30 min. PI-stained cells were analyzed with a FACSverse cytometer (BD Bioscience, Franklin Lakes, NJ, USA).

### Preparation of conditioned media

shCTRL and shRUNX3 Ca9.22 cells (1 × 10^5^ cells/dish) were seeded in 100-mm culture dishes for 24 h. The culture medium was changed to serum-free DMEM/F12, and the cells were treated with TGF-β for 24 h. The culture media were collected and centrifuged at 500 × g for 5 min. The supernatant served as the conditioned medium for subsequent experiments.

### Western blot analysis

shCTRL and shRUNX3 Ca9.22 cells (1 × 10^5^ cells/dish) were seeded in 100-mm culture dishes and incubated with or without TGF-β at 37°C for 24 h. hFOB1.19 cells (1 × 10^6^ cells/dish) were incubated for 6 h with 70% conditioned media obtained from shCTRL and shRUNX3 Ca9.22 cells. The total cell lysates were prepared using RIPA buffer containing protease inhibitor cocktail (Roche Diagnostics, Penzberg, Germany) and centrifuged at 22,000 × g for 15 min at 4°C. The cytoplasmic and nuclear protein extracts were prepared using a nuclear/cytosol fractionation kit (BioVision, Mountain View, CA, USA). The target proteins were detected with 1:1,000 dilutions of the corresponding primary antibodies as described previously [36].

### Gelatin zymography

shCTRL and shRUNX3 Ca9.22 cells (1 × 10^5^ cells/dish) were seeded in 100-mm culture dishes and incubated with or without 10 ng/ml TGF-β for 24 h. The culture media were collected by centrifugation at 200 × g for 5 min. The media were electrophoresed in a 8% polyacrylamide gel containing 0.8 mg/ml gelatin. The gels were washed with 2.5% Triton X-100 for 1 h at room temperature and incubated in a reaction buffer containing 50 mM Tris-HCl (pH 7.5), 5 mM CaCl_2_, 200 mM NaCl and 0.02% Brij35 at 37°C for 24 h. The gels were then stained with 0.1% Coomassie Blue R-250, and the gelatinase activities of MMP-2 and MMP-9 were determined based on the clear bands against the Coomassie-stained background.

### Immunofluorescence staining and confocal imaging

shCTRL and shRUNX3 Ca9.22 cells (1 × 10^3^ cells/well) were seeded into a chamber slide and incubated in complete media for 24 h with or without TGF-β (10 ng/ml). The cells were fixed with 4% paraformaldehyde (w/v), and permeabilized with Triton X-100 buffer. After blocking with 2% goat serum in PBS, the cells were incubated with anti-E-cadherin (BD Biosciences) and anti-β-catenin (Santa Cruz Biotechnology) overnight at 4°C. After washing, the cells were incubated with Alexa Fluor 488 goat anti-mouse IgG and Alexa Fluor 594 goat anti-rabbit IgG (Invitrogen) for 1 h at room temperature. The slide was mounted using Vectashield mounting medium with DAPI (Vector Laboratories, CA, USA). The images were collected using a Zeiss LSM 700 confocal microscope.

### Enzyme-linked immunosorbent assay

The secreted levels of PTHrP, RANKL, and OPG were measured with enzyme-linked immunosorbent assay (ELISA). shCTRL or shRUNX3 Ca9.22 cells (1 × 10^3^ cells/well) were seeded into a 96-well plate and incubated for 24 h with or without TGF-β (10 ng/ml). The culture media were used for the measurement of secreted PTHrP level using commercially available ELISA kit (EIAab, Wuhan, China) according to the manufacturer's recommendations. hFOB1.19 cells (1 × 10^4^ cells/well) were seeded into a 96-well plate and incubated for 24 h with conditioned media from shCTRL or shRUNX3 Ca9.22 cells stimulated with or without TGF-β. The culture media from hFOB1.19 cells were analyzed for the secreted levels of RANKL and OPG using RANKL ELISA kit (EIAab, Wuhan, China) and OPG ELISA kit (Boster Biological Technology, CA, USA) according to the manufacturer's instructions.

### Public database analysis

The head and neck dataset from TCGA Research Network (http://cancergenome.nih.gov/) was analyzed for RUNX3, TGF-β, and PTHrP expression. Kaplan–Meier survival analysis was performed using SPSS 23 (IBM SPSS, Armonk, NY, USA). The patient samples have been split into higher and lower expression groups using the following setting of best cutoff. *P* values were calculated by a log rank test.

### Statistical analyses

The results are expressed as the mean ± standard error (SE) and were analyzed with a one-way analysis of variance (ANOVA) and Student's *t-test* to express the differences between two groups. Dot plots were presented using GraphPad Prism 7 (GraphPad Software, San diego, CA, USA). A *p* value < 0.05 was considered significant.

## SUPPLEMENTARY MATERIALS FIGURES



## References

[1] Jemal A, Bray F, Center MM, Ferlay J, Ward E, Forman D (2011). Global cancer statistics. CA Cancer J Clin.

[2] Siegel R, Ma J, Zou Z, Jemal A (2014). Cancer statistics. CA Cancer J Clin.

[3] Howlader N, Noone AM, Krapcho M, Garshell J, Miller D, Altekruse S, Kosary C, Yu MR, Ruhl J, Tatalovich Z, Mariotto A, Lewis DR, Chen HS (1975–2012). SEER Cancer Statistics Review.

[4] Jimi E, Furuta H, Matsuo K, Tominaga K, Takahashi T, Nakanishi O (2011). The cellular and molecular mechanisms of bone invasion by oral squamous cell carcinoma. Oral Dis.

[5] Ledeboer QC, Velden LA, Boer MF, Feenstra L, Pruyn JF (2005). Physical and psychosocial correlates of head and neck cancer: an update of the literature and challenges for the future (1996–2003). Clin Otolaryngol.

[6] Monnier Y, Broome M, Betz M, Bouferrache K, Ozsahin M, Jaques B (2011). Mandibular osteoradionecrosis in squamous cell carcinoma of the oral cavity and oropharynx: incidence and risk factors. Otolaryngol Head Neck Surg.

[7] Juarez P, Guise TA (2011). TGF-beta in cancer and bone: implications for treatment of bone metastases. Bone.

[8] Nakamura R, Kayamori K, Oue E, Sakamoto K, Harada K, Yamaguchi A (2015). Transforming growth factor-beta synthesized by stromal cells and cancer cells participates in bone resorption induced by oral squamous cell carcinoma. Biochem Biophys Res Commun.

[9] Siegel PM, Massague J (2003). Cytostatic and apoptotic actions of TGF-beta in homeostasis and cancer. Nat Rev Cancer.

[10] Goda T, Shimo T, Yoshihama Y, Hassan NM, Ibaragi S, Kurio N, Okui T, Honami T, Kishimoto K, Sasaki A (2010). Bone destruction by invading oral squamous carcinoma cells mediated by the transforming growth factor-beta signaling pathway. Anticancer Res.

[11] Ito Y, Miyazono K (2003). RUNX transcription factors as key targets of TGF-beta superfamily signaling. Curr Opin Genet Dev.

[12] Li QL, Ito K, Sakakura C, Fukamachi H, Inoue K, Chi XZ, Lee KY, Nomura S, Lee CW, Han SB, Kim HM, Kim WJ, Yamamoto H (2002). Causal relationship between the loss of RUNX3 expression and gastric cancer. Cell.

[13] Chuang LS, Ito Y (2010). RUNX3 is multifunctional in carcinogenesis of multiple solid tumors. Oncogene.

[14] Subramaniam MM, Chan JY, Yeoh KG, Quek T, Ito K, Salto-Tellez M (2009). Molecular pathology of RUNX3 in human carcinogenesis. Biochim Biophys Acta.

[15] Tanji Y, Osaki M, Nagahama Y, Kodani I, Ryoke K, Ito H (2007). Runt-related transcription factor 3 expression in human oral squamous cell carcinomas; implication for tumor progression and prognosis. Oral Oncol.

[16] Gao F, Huang C, Lin M, Wang Z, Shen J, Zhang H, Jiang L, Chen Q (2009). Frequent inactivation of RUNX3 by promoter hypermethylation and protein mislocalization in oral squamous cell carcinomas. J Cancer Res Clin Oncol.

[17] Supic G, Kozomara R, Jovic N, Zeljic K, Magic Z (2011). Hypermethylation of RUNX3 but not WIF1 gene and its association with stage and nodal status of tongue cancers. Oral Oncol.

[18] Kudo Y, Tsunematsu T, Takata T (2011). Oncogenic role of RUNX3 in head and neck cancer. J Cell Biochem.

[19] Tsunematsu T, Kudo Y, Iizuka S, Ogawa I, Fujita T, Kurihara H, Abiko Y, Takata T (2009). RUNX3 has an oncogenic role in head and neck cancer. PLoS One.

[20] Quan J, Johnson NW, Zhou G, Parsons PG, Boyle GM, Gao J (2012). Potential molecular targets for inhibiting bone invasion by oral squamous cell carcinoma: a review of mechanisms. Cancer Metastasis Rev.

[21] Hwang YS, Zhang X, Park KK, Chung WY (2013). An orthotopic and osteolytic model with a newly established oral squamous cell carcinoma cell line. Arch Oral Biol.

[22] Choi S, Myers JN (2008). Molecular pathogenesis of oral squamous cell carcinoma: implications for therapy. J Dent Res.

[23] Scanlon CS, EA Van Tubergen, Inglehart RC, D’Silva NJ (2013). Biomarkers of epithelial-mesenchymal transition in squamous cell carcinoma. J Dent Res.

[24] Quan J, Elhousiny M, Johnson NW, Gao J (2013). Transforming growth factor-beta1 treatment of oral cancer induces epithelial-mesenchymal transition and promotes bone invasion via enhanced activity of osteoclasts. Clin Exp Metastasis.

[25] Soki FN, Park SI, McCauley LK (2012). The multifaceted actions of PTHrP in skeletal metastasis. Future Oncol.

[26] Rao LP, Das SR, Mathews A, Naik BR, Chacko E, Pandey M (2004). Mandibular invasion in oral squamous cell carcinoma: investigation by clinical examination and orthopantomogram. Int J Oral Maxillofac Surg.

[27] Pandey M, Rao LP, Das SR (2009). Predictors of mandibular involvement in cancers of the oromandibular region. J Oral Maxillofac Surg.

[28] Guise TA, Mundy GR (1998). Cancer and bone. Endocr Rev.

[29] Coleman RE (2011). Bone cancer in 2011: Prevention and treatment of bone metastases. Nat Rev Clin Oncol.

[30] Totsuka Y, Usui Y, Tei K, Fukuda H, Shindo M, Iizuka T, Amemiya A (1991). Mandibular involvement by squamous cell carcinoma of the lower alveolus: analysis and comparative study of histologic and radiologic features. Head Neck.

[31] Wong RJ, Keel SB, Glynn RJ, Varvares MA (2000). Histological pattern of mandibular invasion by oral squamous cell carcinoma. Laryngoscope.

[32] Dougall WC, Chaisson M (2006). The RANK/RANKL/OPG triad in cancer-induced bone diseases. Cancer Metastasis Rev.

[33] Sato K, Lee JW, Sakamoto K, Iimura T, Kayamori K, Yasuda H, Shindoh M, Ito M, Omura K, Yamaguchi A (2013). RANKL synthesized by both stromal cells and cancer cells plays a crucial role in osteoclastic bone resorption induced by oral cancer. Am J Pathol.

[34] Tay JY, Bay BH, Yeo JF, Harris M, Meghji S, Dheen ST (2004). Identification of RANKL in osteolytic lesions of the facial skeleton. J Dent Res.

[35] Jun AY, Kim HJ, Park KK, Son KH, Lee DH, Woo MH, Chung WY (2014). Tetrahydrofurofuran-type lignans inhibit breast cancer-mediated bone destruction by blocking the vicious cycle between cancer cells, osteoblasts and osteoclasts. Invest New Drugs.

[36] Kim KR, Kim HJ, Lee SK, Ma GT, Park KK, Chung WY (2015). 15-deoxy-Δ12,14-prostaglandin j2 inhibits osteolytic breast cancer bone metastasis and estrogen deficiency-induced bone loss. PLoS One.

