# Supplementary material for: Loss of RUNX3 expression inhibits bone invasion of oral squamous cell carcinoma
Source: Oncotarget. 2016 Dec 21;8(6):9079–92. doi: 10.18632/oncotarget.14071 (PMC5354716; doi:10.18632/oncotarget.14071)
Supplement: Supplementary file 1 [file oncotarget-08-9079-s001.pdf]

Loss of RUNX3 expression inhibits bone invasion of oral squamous cell carcinoma

Supplementary Materials

MATERIALS AND METHODS

Cell culture

HSC2 cells derived from human mouth squamous cell carcinoma (SCC) and HSC3 cells from human tongue SCC were purchased from the Japanese Collection of Research Bioresources Cell Bank (Shinjuku, Japan). YD10B cells derived from human tongue SCC were obtained from the Department of Oral pathology, College of Dentistry, Yonsei University (Seoul, Korea). The cells were grown in the same condition with Ca9.22 cells.

siRNA-mediated transient knockdown of RUNX3

A RUNX3-targeting siRNA duplex was introduced into YD10B cells using Lipofectamine RNAiMAX (Invitrogen Life Technologies, Carlsbad, CA, USA) in a 24-well plate. RNAi duplex and RNAiMAX were mixed at a ratio of 6 (pmol):1 (μl) in Opti-MEM and incubated for 20 minutes at room temperature. YD10B cells (1×10^3 cells/well) in complete medium without antibiotics were added and incubated for 48 h. The cells were used for further experiments.

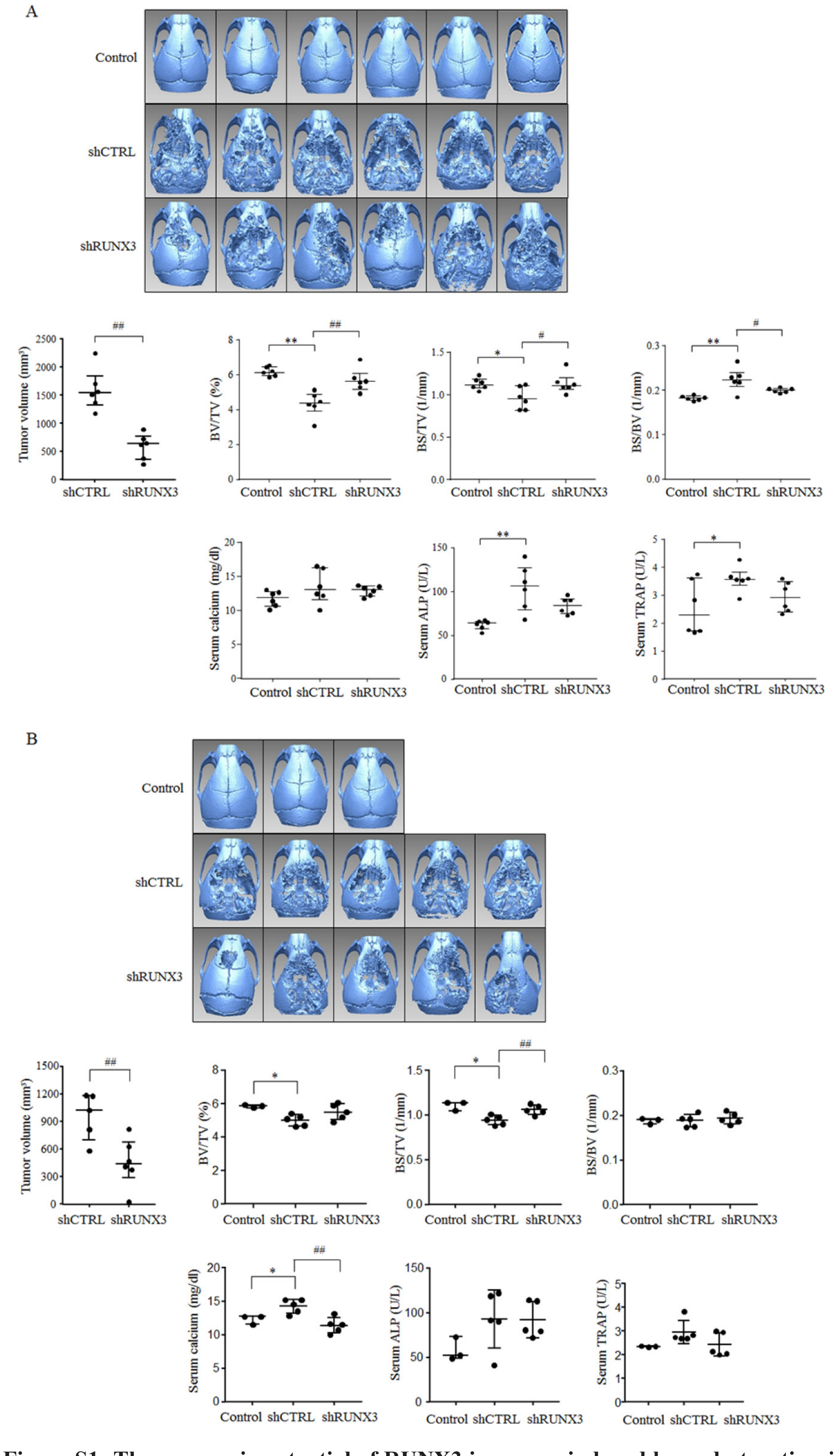

Supplementary Figure S1: The oncogenic potential of RUNX3 in cancer-induced bone destruction *in vivo*. shCTRL or shRUNX3 Ca9.22 OSCC cells (1×10^7 cells/100 µl of HBSS) were subcutaneously injected at the mouse calvaria (**A**: $N = 6$, **B**: $N = 5$). Control mice (A: $N = 6$, B: $N = 3$) were injected with HBSS only. On day 28, two-dimensional (2D) images of the collected carvaria were generated from the µCT data using the NRrecon software, and 3D images were reconstructed from 2D images with the rapidform2006 software. BV/TV (%), BS/TV (1/mm), and BS/BV (1/mm) served as bone morphometric parameters of the calvaria were determined using the µCT images. Serum levels of the bone turnover markers Ca^{2+}, ALP, and TRAP5b were estimated using kits as described in the Materials and Methods. The results are expressed as the median with interquartile range of 6 mice per group. $^{*}P < 0.05$, $^{**}P < 0.01$ versus HBSS-injected control mice, $^{\#}P < 0.05$, $^{\#\#}P < 0.01$ versus shCTRL cell-inoculated mice.

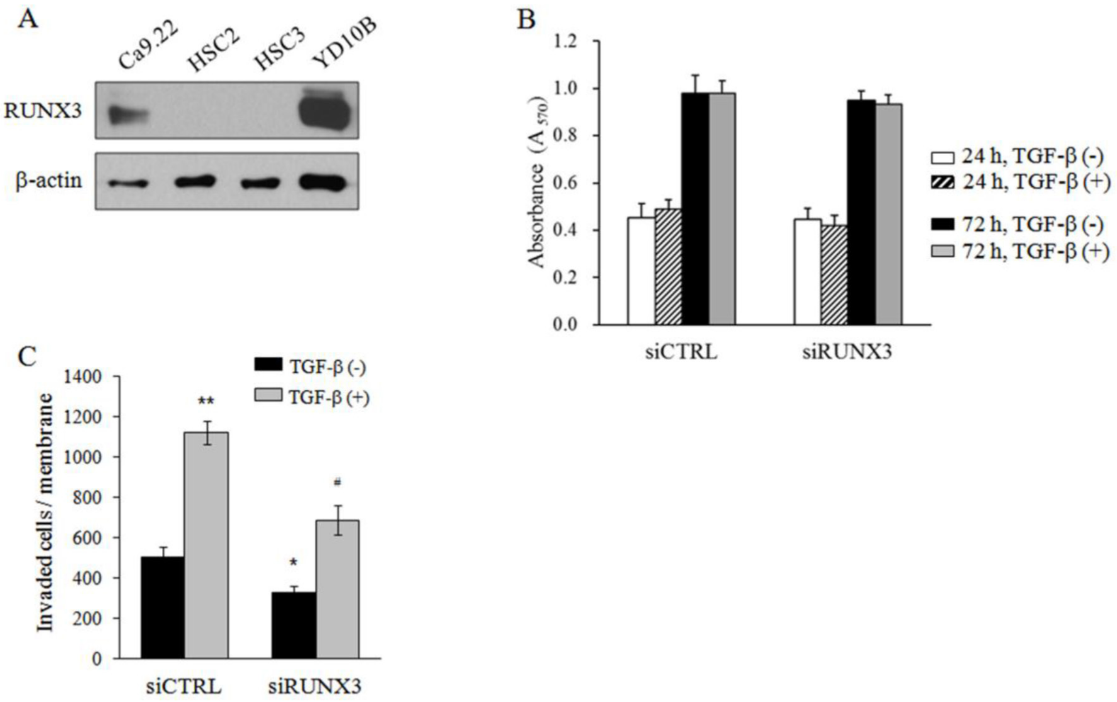

Supplementary Figure S2: The effect of RUNX3 knockdown on the viability and invasion of YD10B OSCC cells. (**A**) RUNX3 expression was confirmed in 4 OSCC cell lines by a Western blot analysis. (**B**) siCTRL or siRUNX3 YD10B cells (1×10^3 cells/well) were treated with 10 ng/ml TGF-β for 24 h or 72 h. Cell viability was determined using an MTT assay. (**C**) siCTRL and siRUNX3 YD10B cells (5×10^4 cells/0.1 ml) were seeded into the upper chamber coated with gelatin and Matrigel. Complete medium containing 5% FBS and 10 ng/ml TGF-β was added to the upper chamber, and the lower chamber was filled with 0.6 ml of complete medium containing 10% FBS. The number of invaded cells was counted as described in Material and methods. The results are expressed as the mean ± SE. $^{*}P < 0.05$, $^{**}P < 0.01$ versus siCTRL cells, $^{\#}P < 0.05$ versus TGF-β-treated siCTRL cells.

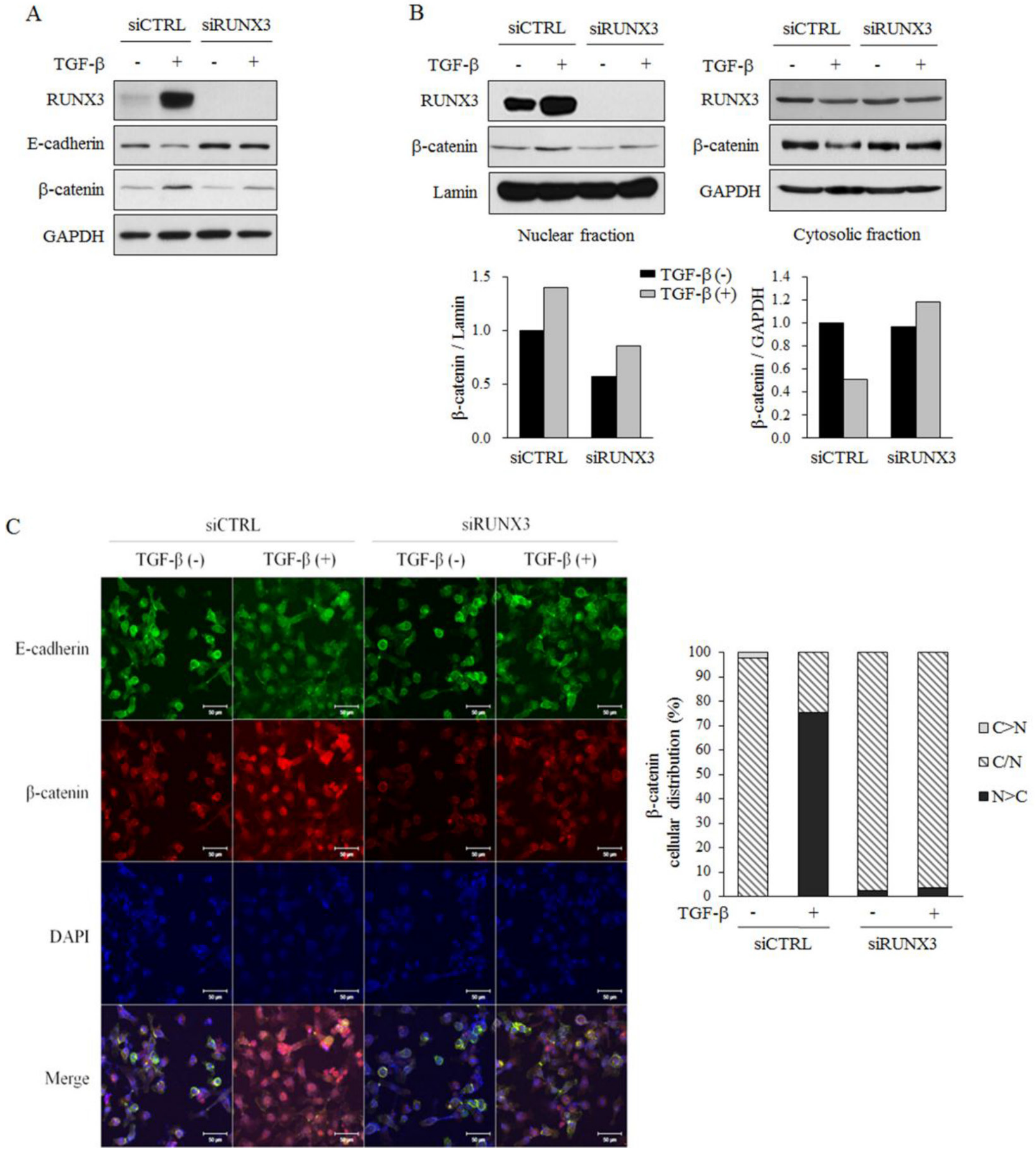

Supplementary Figure S3: The effect of RUNX3 knockdown on the expression of EMT markers in YD10B OSCC cells. siCTRL or siRUNX3 cells (1×10^5 cells/dish) were treated with 10 ng/ml TGF-β for 24 h. (**A**) The expression levels of RUNX3, E-cadherin, and β-catenin were examined in the total lysates with a Western blot analysis. GAPDH served as a loading control. (**B**) The levels of RUNX3 and β-catenin in cytoplasmic and nuclear protein extracts were determined via Western blot analysis. Lamin served as a loading control for the nuclear fraction. The images are representative of three independent experiments. The graphs illustrate the ratio of the densitometric intensity of β-catenin after normalization to lamin or GAPDH. (**C**) Subcellular localization of E-cadherin and β-catenin was examined by confocal microscopy as described in Materials and methods. Cellular distribution of β-catenin was shown as percentage of siCTRL or siRUNX3 cells with its cytoplasmic (C > N), cytoplasmic and nuclear (C/N), and nuclear (N > C) localization.

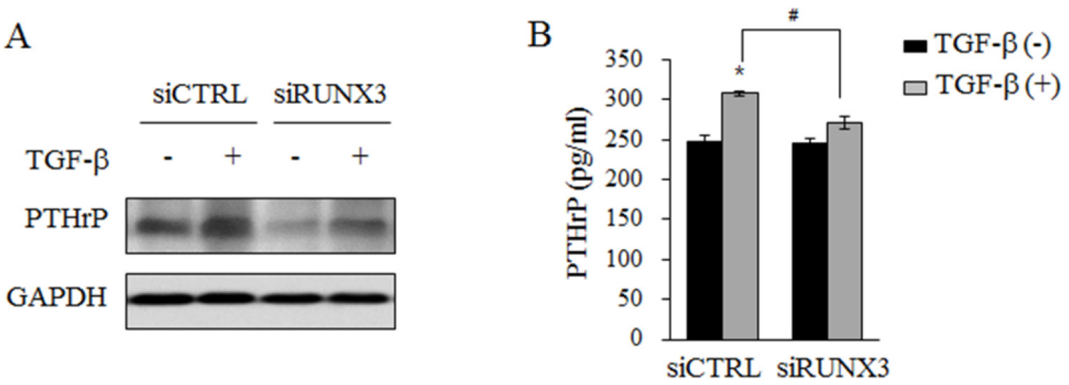

Supplementary Figure S4: The effect of RUNX3 knockdown on the expression of PTHrP in YD10B OSCC cells. siCTRL or siRUNX3 YD10B cells (1×10^5 cells/dish) were treated with 10 ng/ml TGF-β for 24 h. (**A**) The expression levels of PTHrP were determined with a Western blot analysis. GAPDH served as a loading control. (**B**) siCTRL or siRUNX3 YD10B cells (1×10^3 cells/well) were treated with 10 ng/ml TGF-β for 24 h. PTHrP levels in the conditioned media were analyzed with an ELISA kit.
